# Tracer kinetic assessment of blood–brain barrier leakage and blood volume in cerebral small vessel disease: Associations with disease burden and vascular risk factors

**DOI:** 10.1016/j.nicl.2021.102883

**Published:** 2021-11-17

**Authors:** Michael S. Stringer, Anna K. Heye, Paul A. Armitage, Francesca Chappell, Maria del C. Valdés Hernández, Stephen D.J. Makin, Eleni Sakka, Michael J. Thrippleton, Joanna M. Wardlaw

**Affiliations:** a Centre for Clinical Brain Sciences, University of Edinburgh, Edinburgh, UK; b UK DRI at the University of Edinburgh, University of Edinburgh, Edinburgh, UK; c Edinburgh Clinical Trials Unit, Usher Institute, University of Edinburgh, Edinburgh, UK; d Academic Unit of Radiology, Department of Infection, Immunity and Cardiovascular Disease, University of Sheffield, Royal Hallamshire Hospital, Sheffield, UK; e Centre for Rural Health, University of Aberdeen, Inverness, UK

**Keywords:** Blood brain barrier, Cerebrovascular disease, MRI, Stroke, SVD

## Abstract

Subtle blood–brain barrier (BBB) permeability increases have been shown in small vessel disease (SVD) using various analysis methods. Following recent consensus recommendations, we used Patlak tracer kinetic analysis, considered optimal in low permeability states, to quantify permeability-surface area product (*PS*), a BBB leakage estimate, and blood plasma volume (*v_P_*) in 201 patients with SVD who underwent dynamic contrast-enhanced MRI scans. We ran multivariable regression models with a quantitative or qualitative metric of white matter hyperintensity (WMH) severity, demographic and vascular risk factors. *PS* increased with WMH severity in grey (B = 0.15, Confidence Interval (CI): [0.001,0.299], p = 0.049) and normal-appearing white matter (B = 0.015, CI: [−0.008,0.308], p = 0.062). Patients with more severe WMH had lower *v_P_* in WMH (B = -0.088, CI: [−0.138,-0.039], p < 0.001), but higher *v_P_* in normal-appearing white matter (B = 0.031, CI: [−0.004,0.065], p = 0.082). *PS* and *v_P_* were lower at older ages in WMH, grey and white matter. We conclude higher *PS* in normal-appearing tissue with more severe WMH suggests impaired BBB integrity beyond visible lesions indicating that the microvasculature is compromised in normal-appearing white matter and WMH. BBB dysfunction is an important mechanism in SVD, but associations with clinical variables are complex and underlying damage affecting vascular surface area may alter interpretation of tracer kinetic results.

## Highlights

• Permeability surface area (*PS*) was higher, even in normal appearing tissue.
• *PS* was higher in patients with more white matter hyperintensities.
• Tissue damage affecting vascular surface area may affect how we interpret tracer kinetic results.

## 1 Introduction

Cerebral small vessel disease (SVD) is an underlying cause in about 25% of strokes and up to 50% of dementias (alone or in combination with Alzheimer’s disease (AD)) (Wardlaw et al., 2019). The effects of SVD on the brain appear as several types of lesions visible on structural MRI, the commonest of which are white matter hyperintensities (WMH) (Wardlaw et al., 2019, Wardlaw et al., 2013).

The vascular dysfunction that leads to SVD brain damage is poorly understood, but dysfunction of the blood–brain barrier (BBB) has been proposed. Various components of BBB dysfunction may cause brain damage including leakage of fluids, proteins and fibrinogen into the vessel walls and perivascular tissues which could lead to thickening and stiffening of the arteriole wall, which may in turn reduce vasoreactivity, oxygen and essential nutrient transport, plus perivascular oedema and inflammation (Wardlaw et al., 2019). BBB leakiness increases subtly with normal ageing (Erdő et al., 2017, Farrall and Wardlaw, 2009), but has also been found in patients with Alzheimer’s disease and in patients with small vessel (lacunar) stroke; leakage was worse in patients with more WMH, and, specifically, worse in WMH (Li et al., 2017, Wardlaw et al., 2017, Zhang et al., 2017). Subtle BBB leakiness may also lead to hippocampal damage and cognitive decline after stroke and in patients at risk of AD (Li et al., 2017, Li et al., 2018, Montagne et al., 2020, Nation et al., 2019, Sweeney et al., 2018, Wardlaw et al., 2017, Zhang et al., 2017).

Several methods have been proposed to evaluate BBB permeability in people, with dynamic contrast-enhanced MRI (DCE-MRI) being most widely used for quantification *in vivo* (Thrippleton et al., 2019). DCE-MRI exploits the paramagnetic properties of gadolinium-based contrast agents to track T1-weighted signal enhancement over time to assess the intra- and extra-vascular distribution of the tracer. Multiple analytical approaches have been employed to measure these changes, however recent literature and recommendations (Raja et al., 2018, Thrippleton et al., 2019) have converged on tracer kinetic modelling to estimate the vascular permeability-surface area product (*PS*) as an estimate of BBB leakage in a given region of interest. This method simultaneously calculates plasma volume fraction (*v_P_*) in the same region of interest. These two values together provide complementary measures of key aspects of vascular dysfunction at tissue level.

Previously, we used a semi-quantitative approach with linear mixed modelling of the signal enhancement slopes to compare relative leakage between tissues, by SVD and patient characteristics, but did not calculate an individual patient or tissue quantitative leakage parameter (Wardlaw et al., 2017). We identified higher leakage in WMH than in normal appearing white matter, with leakage in normal appearing white matter also increasing with proximity to WMH and with WMH burden (Wardlaw et al., 2017). While semi-quantitative approaches can be implemented easily, they do not provide quantitative markers of BBB leakage, limiting direct comparisons between subjects and studies (Thrippleton et al., 2019). We therefore set out to re-analyse the data using the potentially more sensitive and specific method of tracer kinetic analysis to confirm the previous findings.

In this work, we performed tracer kinetic modelling according to recent consensus recommendations (Thrippleton et al., 2019), analysed since the original semi-quantitative results were published from the same dataset (Wardlaw et al., 2017), and assessed associations between disease related and demographic variables with *PS* and *v_P_* in grey matter, white matter, WMH and recent stroke lesions. We hypothesised that *PS* in grey matter, white matter and WMH would increase with WMH burden, and that *v_P_* would correspondingly decrease in WMH and surrounding healthy tissue due to impaired cerebral blood flow.

## 2 Methods

### 2.1 Recruitment

The inclusion criteria, participant recruitment, medical assessment and MRI acquisition have been published in detail previously (Heye et al., 2016, Valdés Hernández et al., 2015, Wardlaw et al., 2017). Briefly, the patients were recruited prospectively with their first clinically evident mild (non-disabling) ischaemic stroke. Patients were only eligible for inclusion where they had a confirmed diagnosis of ischaemic stroke, capacity to consent, were aged over 18 years and willing to undergo a baseline MRI scan followed by DCE-MRI 1 to 3 months post-stroke. Medical history was obtained by a stroke physician. Stroke subtype (lacunar or cortical) was determined by a panel of stroke experts based on clinical and MRI features (Wardlaw et al., 2017). The study was approved by the Lothian Ethics of Medical Research Committee (REC 09/81101/54) and NHS Lothian R&D Office (2009/W/NEU/14) and all patients gave written informed consent. The study was conducted in full conformity with the Declaration of Helsinki.

### 2.2 MRI protocol

As previously published (Valdes Hernandez et al., 2015), we performed MRI on a 1.5T GE Signa HDxt scanner with an 8-channel phased array head-coil, and acquired T1-weighted, T2-weighted, FLAIR, gradient echo and diffusion tensor images (Wardlaw et al., 2013). We performed DCE-MRI (Heye et al., 2016) at 1–3 months after stroke to avoid the acute effects of the index stroke on the BBB. Pre-contrast *T*_1_ (*T*_10_) maps were generated from two 3D T1-weighted fast-spoiled gradient-echo (FSPGR) acquisitions with flip angles of 2 and 12° (TR/TE = 8.24/3.1 ms, 24x24cm FOV, 256 × 192 acquisition matrix and 42 × 4 mm thick slices). A 0.2 ml/kg (i.e., 0.1 mmol/kg body weight) dose of Gadoterate meglumine (Gd-DOTA, DOTAREM; Guerbet, Paris, France) was injected intravenously at 2 ml/second via an injection pump. The 3D T1-weighted sequence (flip angle = 12°) was then repeated 20 times sequentially for c.24 min with a temporal resolution of around 73 s, employing a long acquisition time to detect subtle BBB leaks (Wardlaw et al., 2017).

### 2.3 Image analysis

An expert neuroradiologist used validated visual scores to quantify WMH (Fazekas score), other SVD features (lacunes, perivascular spaces, microbleeds, brain atrophy), the recent ‘index’ lacunar or cortical infarct and any old infarcts. We registered structural images to the first pre-contrast FSPGR-12° scan (Jenkinson and Smith, 2001). We generated masks for the intracranial volume, cerebrospinal fluid (CSF), WMH and normal-appearing WM using a validated multispectral method (Valdes Hernandez et al., 2010) before manual editing to remove incorrectly classified tissue. We computationally generated subcortical/deep grey matter masks (Heye et al., 2016). We defined the recent ‘index’ stroke lesion as hyperintense on the diffusion weighted image obtained at diagnosis, including corresponding signal changes on other sequences, and manually masked the lesion using Analyze 11.0 (AnalyzeDirect).

### 2.4 Tracer kinetic modelling

The analysis procedure for the DCE-MRI data was as described in Heye et al (Heye et al., 2016) except that fitting to the Patlak model was implemented via a multiple linear regression approach, and in line with recent consensus recommendations (Thrippleton et al., 2019). We applied modelling in regions of interest for each of four tissue classes: normal appearing white matter (WM), deep grey matter (GM), white matter hyperintensities (WMH) and recent stroke lesions (RSL). In brief, we calculated the median signal intensity (*S_i_*) across all voxels within each tissue mask and measured signal enhancement (*E_i_*) relative to the corresponding pre-contrast signal intensity (*S_0_*) as *E_i_=(S_i_-S_0_)/S_0_*. We generated *T*_1_ maps using the variable flip angle method (Brookes et al., 1999) before calculating the contrast agent concentration, *C_i_*, as in Armitage et al (Armitage et al., 2005). We determined the vascular input function by manually selecting voxels in the superior sagittal sinus and converted the measured whole-blood concentration *C_b_(t)* to blood plasma concentration *C_p_(t)* using individual haematocrit values as previously described (Heye et al., 2016).

We applied the Patlak model to fit the concentration of contrast agent in the tissue, *C*_t_*(t),* using publicly available in-house software (https://github.com/mjt320/DCE-functions) programmed in MATLAB (MathWorks, Natick, MA, USA), as this model has been shown to be most appropriate for assessing low-permeability tissue at low temporal resolution (Barnes et al., 2016, Cramer and Larsson, 2014, Heye et al., 2016), to obtain values for the fractional plasma volume (*v_p_*, 10^−2^) and permeability surface area product (*PS,* 10^−4^ min^−1^). We reported leakage results as estimated *PS* instead of the alternative volume transfer constant, *K^Trans^* symbol, which represents the rate of transport of GBCA from arterial blood plasma to the EES; provided early data points are excluded from the analysis (as here) and the Patlak model assumptions are valid (as previously demonstrated (Barnes et al., 2016, Cramer and Larsson, 2014, Heye et al., 2016)), the arterial, capillary and venous GBCA blood plasma concentrations are similar and *K*^Trans^≈*PS*. For comparison we also reported the signal enhancement slopes (signal intensity change per unit time) previously calculated in the same dataset (Munoz Maniega et al., 2017).

### 2.5 Statistics

We constructed multiple linear regression models in R (v3.6.3) to explore the relationship between the permeability surface area (*PS*), fractional plasma volume (*v_P_*) and WMH burden (assessed as a) Fazekas score (0–6, summing the deep and periventricular white matter components) and b) WMH volume normalised to the intracranial volume in separate models), accounting for age, stroke subtype (cortical (0) vs lacunar (1)), mean arterial blood pressure, hypertension diagnosis (diagnosed (1), not diagnosis (0)), pulse pressure and smoking status (current/stopped within last year (1), never/stopped over a year (0)) to control for key vascular risk factors and for consistency with the previous semi-quantitative analysis (Wardlaw et al., 2017). We included results for models including Fazekas score and WMH volume normalised to the intracranial volume, as while highly correlated visual and computational measures of WMH severity differ (Valdes Hernandez et al., 2013), they may provide complementary information. We checked normality of residuals and homogeneity of variance using Q-Q plots, histograms and plots of residuals vs fitted values for each model. We checked variance inflation factors for each model to avoid multi-collinearity.

To explore whether observed associations may have been influenced by differences in the vascular input function associated with age or WMH burden, we dichotomised by median Fazekas score (3) for high (>=4)/low (<=3) Fazekas score and above/below median age (66.72 years). We assessed the peak and slope of the Gd-time curves by plotting the mean vascular input function and Gd concentration over time in each tissue type, including indicators of standard mean errors and standard deviation.

## 3 Results

DCE-MRI data suitable for analysis were obtained in 201 patients, with a median age of 66.8 (Interquartile range: 56.8–75.2) years, on average 38 days after stroke. Key demographic and clinical parameters are provided in Table 1 along with summary statistics for the DCE-MRI parameters. PS was highest in recent stroke lesions, followed by WMH, GM and WM. Variance inflation factors were below 2 for all included variables in each model.

**Table 1:** Patient demographics of the 201 patients with DCE-MRI data. Categorical data are presented as n (%), for continuous variables mean (standard deviation) is given unless specified. IQR = Inter-quartile range.

**Demographic/clinical risk factors**		
Age (IQR)		66.7 (56.8–75.2)
Stroke subtype	Lacunar	92 (45.8%)
	Cortical	109 (54.2%)
Mean arterial pressure median (IQR)		141 (130–159)
Hypertension		150 (73.1%)
Pulse pressure median (IQR)		60 (49–76)
Smoking status (current or stopped in last year)		72 (35.8%)

**White matter burden parameters**		
Fazekas score	0	7 (3.4%)
	1	17 (8.5%)
	2	74 (36.8%)
	3	23 (11.4%)
	4	29 (14.4%)
	5	20 (10.0%)
	6	31 (15.4%)
% WMH in intracranial volume, median (IQR)		0.91 (0.31–2.36)

**DCE-MRI parameters** (*PS*: 10^−4^ min^−1^, *v_P_*: 10^−2^, Signal enhancement slope: % min^−1^)		
Deep grey matter	*PS*	3.896 (1.588)
	*v_P_*	1.232 (0.406)
	*Signal enhancement slope*	0.00533 (0.05096)
Normal-appearing white matter	*PS*	2.929 (1.672)
	*v_P_*	0.600 (0.370)
	*Signal enhancement slope*	0.02215 (0.03969)
White matter hyperintensities	*PS*	3.941 (1.775)
	*v_P_*	0.825 (0.560)
	*Signal enhancement slope*	0.03107 (0.05564)
Recent stroke lesions	*PS*	5.714 (4.700)
	*v_P_*	0.815 (0.584)
	*Signal enhancement slope*	0.08518 (0.11335)

### 3.1 Associations between tracer kinetic parameters, clinical and SVD imaging variables

*PS* decreased with age in WM (B = -0.044, CI: [−0.07, −0.018]), GM (B = -0.044, CI: [−0.068, −0.019]) and WMH (B = -0.03, CI: [−0.058, −0.002]) but not in the recent infarct (B = 0.019, CI: [−0.067, 0.105]). There was no association of *PS* with hypertension or smoking in GM, WM or WMH (Table 2, Supplementary Table 1), but *PS* was higher in the recent infarct in patients with versus without hypertension (B = 2.195, CI: [0.194, 4.197]).

**Table 2:** Permeability surface area (*PS*, 10^−4^ min^−1^) against age (years), Fazekas score, stroke subtype, mean arterial pressure (mmHg), hypertension status, pulse pressure (mmHg) and smoking status in each tissue type of interest. (WM = white matter, CI = confidence interval, MAP = mean arterial pressure).

	Tissue *PS* associations using Fazekas score			
Tissue	Variable	B coefficient	95% CI	p-value
Normal appearing WM	Age	−0.044	−0.070 to −0.018	*<0.001*
	Fazekas score	0.150	−0.008 to 0.308	*0.062*
	Stroke subtype	−0.398	−0.868 to 0.072	*0.097*
	MAP	−0.006	−0.023 to 0.011	0.500
	Hypertension	0.411	−0.141 to 0.963	0.144
	Pulse pressure	0.004	−0.009 to 0.017	0.562
	Smoking status	−0.423	−0.942 to 0.096	0.109
WM Hyperintensities	Age	−0.030	−0.058 to −0.002	*0.035*
	Fazekas score	0.092	−0.081 to 0.265	0.296
	Stroke subtype	−0.039	−0.550 to 0.473	0.882
	MAP	0.002	−0.017 to 0.021	0.862
	Hypertension	0.309	−0.294 to 0.912	0.314
	Pulse pressure	0.002	−0.013 to 0.016	0.810
	Smoking status	−0.028	−0.594 to 0.537	0.922
Grey matter	Age	−0.044	−0.068 to −0.019	*<0.001*
	Fazekas score	0.150	0.001 to 0.299	*0.049*
	Stroke subtype	−0.285	−0.729 to 0.159	0.207
	MAP	−0.004	−0.020 to 0.013	0.650
	Hypertension	0.207	−0.315 to 0.729	0.434
	Pulse pressure	0.010	−0.002 to 0.023	0.103
	Smoking status	0.148	−0.343 to 0.638	0.553
Recent stroke lesion	Age	0.019	−0.067 to 0.105	0.666
	Fazekas score	−0.067	−0.596 to 0.461	0.801
	Stroke subtype	−2.017	−3.621 to −0.413	*0.014*
	MAP	0.027	−0.032 to 0.086	0.366
	Hypertension	2.195	0.194 to 4.197	*0.032*
	Pulse pressure	−0.020	−0.064 to 0.025	0.385
	Smoking status	0.774	−0.965 to 2.514	0.380

**Fig. 1:**
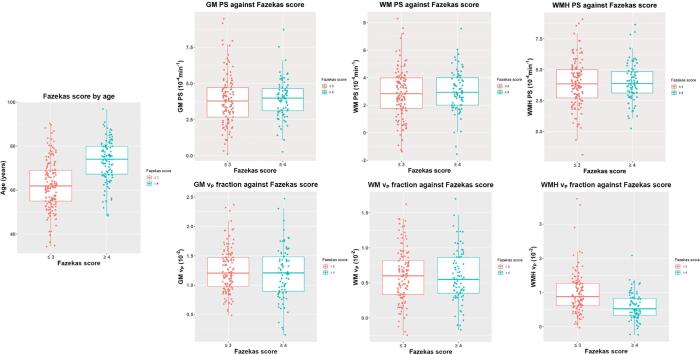
Plots of dichotomised Fazekas score (≤3 in pink and ≥4 in blue) against age, permeability surface area (*PS*), top, and blood plasma volume fraction (*v_P_*), bottom, in grey matter (GM), white matter (WM) and white matter hyperintensities (WMH). (For interpretation of the references to colour in this figure legend, the reader is referred to the web version of this article.)

*PS* increased with the severity of WMH (degree of white matter injury) in GM (B = 0.150, CI: [0.001, −0.299]) with a trend in WM (B = 0.150, CI: [−0.008, 0.308]) but not in WMH (B = 0.092, CI: [−0.081, 0.265]) or in the recent infarct (B = -0.067, CI: [−0.596, 0.461]) (Fig. 1). Associations were stronger for WMH severity expressed as Fazekas score than for WMH volume (Table 2, Supplementary Table 1). Also, *PS* was higher in recent cortical than lacunar infarcts (B = -2.017, CI: [−3.621, −0.413]).

*v_P_* decreased with age in WM (B = -0.008, CI: [−0.013, −0.002]), GM (B = -0.007, CI: [−0.014, −0.001]), and WMH (B = -0.008, CI: [−0.016, 0.000]) but not in the recent infarct (B = -0.001, CI: [−0.012, 0.010]) (Table 3, Supplementary Table 2). Additionally, *v_P_* declined with hypertension in GM (B = -0.145, CI: [−0.279, −0.012]) and WM (B = -0.162, CI: [−0.283, −0.040]) and with smoking in GM (B = -0.131, CI: [−0.256, −0.006]).

**Table 3:** Plasma volume fraction (*v_P_*, 10^−2^) against age (years), Fazekas score, stroke subtype, mean arterial pressure (mmHg), hypertension status, pulse pressure (mmHg) and smoking status in each tissue type of interest. (WM = white matter, CI = confidence interval, MAP = mean arterial pressure).

	Tissue v_P_ associations using Fazekas score			
Tissue	Variable	B coefficient	95% CI	p-value
Normal appearing WM	Age	−0.008	−0.013 to −0.002	*0.008*
	Fazekas score	0.031	−0.004 to 0.065	*0.082*
	Stroke subtype	0.060	−0.043 to 0.163	0.255
	MAP	0.001	−0.003 to 0.005	0.648
	Hypertension	−0.162	−0.283 to −0.040	*0.009*
	Pulse pressure	−0.000	−0.003 to 0.003	0.969
	Smoking status	−0.101	−0.215 to 0.013	*0.081*
WM Hyperintensities	Age	−0.008	−0.016 to 0.000	*0.063*
	Fazekas score	−0.088	−0.138 to −0.039	*<0.001*
	Stroke subtype	0.078	−0.069 to 0.225	0.295
	MAP	0.001	−0.004 to 0.006	0.718
	Hypertension	−0.172	−0.346 to 0.001	*0.051*
	Pulse pressure	−0.001	−0.005 to 0.003	0.662
	Smoking status	−0.002	−0.164 to 0.161	0.985
Grey matter	Age	−0.007	−0.014 to −0.001	*0.020*
	Fazekas score	0.010	−0.028 to 0.048	0.614
	Stroke subtype	0.073	−0.041 to 0.186	0.207
	MAP	−0.000	−0.004 to 0.004	0.917
	Hypertension	−0.145	−0.279 to −0.012	*0.033*
	Pulse pressure	−0.001	−0.004 to 0.003	0.708
	Smoking status	−0.131	−0.256 to −0.006	*0.041*
Recent stroke lesion	Age	−0.001	−0.012 to 0.010	0.887
	Fazekas score	−0.026	−0.093 to 0.041	0.449
	Stroke subtype	−0.136	−0.339 to 0.068	0.189
	MAP	0.001	−0.006 to 0.009	0.755
	Hypertension	−0.159	−0.412 to 0.095	0.218
	Pulse pressure	0.001	−0.004 to 0.007	0.644
	Smoking status	0.091	−0.130 to 0.311	0.418

*v_P_* declined with increasing WMH severity in WMH (B = -0.088, CI: [−0.138, −0.039]) but increased in WM (B = 0.031, CI: [−0.004, 0.065]), no effect was present in GM (B = 0.010, CI: [−0.028, 0.058]) or the recent infarct (B = -0.026, CI: [−0.093, 0.041]) (Table 3, Supplementary Table 2, Fig. 1). The associations were stronger for WMH severity expressed as volume than for Fazekas score in WM and GM but in WMH associations were stronger for Fazekas score than volume (Table 3, Supplementary Table 2). There was no association between *v_P_* and vascular risk factors in the recent infarct (Table 3, Supplementary Table 2).

### 3.2 Impact of age and WMH severity on vascular input functions and tissue concentration curves

**Fig. 2:**
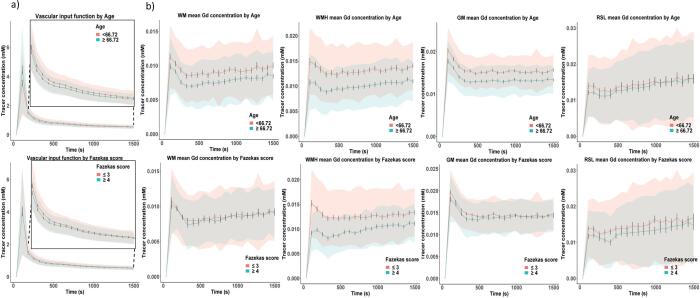
Graphs showing a) mean blood plasma contrast agent concentration across time (vascular input function) measured in the superior sagittal sinus dichotomised by median age (≤66.72 in pink and ≥66.72 in blue, top row) and Fazekas score (≤3 in pink and ≥4 in blue, bottom row) following intravenous (IV) controlled injection of Gadolinium-based contrast agent (Gd) with an inset closeup of the post-bolus section of the curve, and b) mean tracer concentrations over time following IV contrast injection of Gd in white matter (WM), grey matter (GM), white matter hyperintensities (WMH) and recent stroke lesions (RSL) dichotomised by age and Fazekas score against time. Standard deviation is indicated by the shading around each line while error bars denote the standard mean error. (For interpretation of the references to colour in this figure legend, the reader is referred to the web version of this article.)

When dichotomised by median age, Gadolinium concentration measured in blood (i.e. the vascular input function) was slightly higher in the younger patient group after the bolus peak; dichotomising by Fazekas scores showed only a marginal difference at the four timepoints immediately after the bolus peak (Fig. 2a). Fig. 2b shows that in younger versus older patients, the Gadolinium concentration *peak* was higher and the curve thereafter remained higher in GM, WM and WMH, with generally clear separation of the outer bounds of the standard mean error (Fig. 2b), with no difference in the recent infarct. The *slopes* of the mean tissue concentration curves were greatest in recent infarcts (consistent with BBB leakage) followed by WMH, then WM and GM, and were similar for the younger and older patients. In patients with higher versus lower Fazekas scores, tracer concentration *peak* was lower, but the subsequent *slope* was greater in WMH. There was no apparent difference between the *peak* of the curves by Fazekas score in GM, WM or recent infarcts, but the *slope* was steepest in recent infarcts followed by WMH in patients with Fazekas score ≥ 4, WM, WMH in patients with Fazekas score ≤ 3 and GM, similar to the analysis dichotomised by patient age.

## 4 Discussion

We show that *PS*, a quantitative measure of BBB leakage, increases with WMH severity in GM and normal-appearing WM, but not WMH, independent of key risk factors. Mean *PS* was highest in stroke lesions, followed by WMH, GM and WM. With increasing WMH severity *v_P_* declines in WMH and increases in WM, but not GM. The strength of association varied between the different tissues sampled and whether the WMH were quantified by visual score or volume, although the overall pattern was consistent. Both *PS* and *v_P_* decreased with age in GM, WM and WMH consistent with loss of small vessels at older ages.

### 4.1 Comparison to semi-quantitative analysis

As in the previously reported analysis using a semi-quantitative BBB leakage parameter (Wardlaw et al., 2017), we found increased *PS* with WMH severity in GM and WM. Previously, we also found increased leakage in WMH with increasing WMH severity, consistent with the observation of a steeper slope of the Gd-enhancement curve in patients with severe vs milder WMH (Fig. 2b). While the tracer kinetic analysis found *PS* in WMH was higher with worse WMH severity the effect was not significant after correcting for other key variables. In recent stroke lesions, we find *PS* was greater in cortical than lacunar infarcts and increased with hypertension*.* By contrast *PS* and *v_P_* decreased with age in WM, GM and WMH, and associations with *PS* were generally weaker than in the previous analysis*.* We found *v_P_* decreased with hypertension in GM, WM and WMH, but not recent stroke lesions. In the semi-quantitative analysis, leakage increased in GM, WM and WMH with hypertension. Although *v_P_* decreased with smoking in GM, no comparable association was revealed by the semi-quantitative analysis. Subject-specific *PS* and semi-quantitative leakage estimates showed a broadly linear relationship (Supplementary Fig. 1), reflecting previous simulation results (Heye et al., 2016). Both approaches identified the highest leakage rates in recent stroke lesions, followed by WMH and WM. However, while GM had the lowest enhancement slope of all the tissues, *PS* values in GM were higher than in WM and similar to those in WMH. As enhancement slope is not a direct measure of BBB leakage, being influenced by multiple factors including *v_P_* (Heye et al., 2016), such inconsistencies are expected.

### 4.2 Associations between tracer kinetic parameters and WMH severity

As we previously reported, *PS* and *v_P_* are higher in WMH than WM (Heye et al., 2016), so increases in *PS* and *v_P_* with WMH severity in normal appearing tissue may suggest apparently healthy tissue is also compromised. This could result from global changes affecting vessel health or as a prelude to further WMH evolution (Alber et al., 2019, Brown and Thore, 2011), consistent with DTI data from other studies (Munoz Maniega et al., 2017). Impaired blood flow, increased blood volume and delayed transit times have been reported previously in WMH (Arba et al., 2017, Østergaard et al., 2016). Cerebral blood flow might increase in early disease stages to compensate for reduced oxygen extraction efficacy (Ostergaard et al., 2016). Hypoxia in compromised but still normal appearing tissue may therefore lead to higher *v_P_* due to increased blood volume in dilated arterioles or venules (Zhang et al., 2020) also interpreted as dysfunctional blood flow (Bailey et al., 2004). Meanwhile, as vascular surface area and cerebral blood volume scale with vessel density (Varatharaj et al., 2019), *v_P_* in WMH decreasing with WMH severity would be consistent with fewer blood vessels in damaged tissue as WMH severity increases. Hence, both findings would further support the role of BBB dysfunction in SVD even beyond visibly damaged tissue.

Several papers have also found permeability increased with disease severity in normal appearing tissues (Li et al., 2017, Topakian et al., 2010, Wardlaw et al., 2017), but fewer explore associations between disease burden and permeability metrics using quantitative techniques while correcting for key covariates (Li et al., 2017, Li et al., 2018, Taheri et al., 2011, Thrippleton et al., 2019). Li et al found *K^Trans^* increased with WMH severity (Li et al., 2017) and total SVD score (Li et al., 2018) in GM, WM and WMH while *v_P_* decreased with WMH severity in GM, WM and WMH (Li et al., 2017) when adjusted for age, sex, and vascular risk factors, but excluded symptomatic stroke patients. Zhang et al reported decreased permeability (*K_i_*) in WMH with increasing WMH severity but found no associations in GM and WM (Zhang et al., 2019) adjusting for age and sex, but not vascular risk factors. Comparing patients to age matched controls, van de Haar et al found higher *K_i_* in GM and lower *v_P_* in WM, GM, and WMH (van de Haar et al., 2016a, van de Haar et al., 2016b), though associations did not survive correction for diabetes and other non-cardiovascular diseases. Zhang et al also found decreased *v_p_* in WMH of patients relative to age- and sex-matched controls (Zhang et al., 2017) in a univariate analysis, but not when adjusted for risk factors. These variations in findings may result from differences in patient population, sample size, methodology and stage of disease, which may skew towards lower/higher disease burdens or be associated with a more or less acute effect. Region selection and disease quantification methods also differed between studies. While small manually defined regions of interest provide consistent sample volume they may be less representative and potentially subject to rater bias, while Freesurfer can fail in up to 20% of stroke datasets (Liew et al., 2020). Different approaches to assessing disease burden may affect the degree and significance of associations. We found *PS*; and *v_p_* were generally more strongly associated with Fazekas score than WMH volume. While Fazekas score and WMH volume are highly correlated, each has limitations. Computational methods may omit subtle hyperintensities (Valdes Hernandez et al., 2013), although the method used here is very sensitive to subtle WMH. Visual scoring has floor and ceiling effects (although the Fazekas score has a wide dynamic range) and may be less sensitive to small differences in WMH severity than WMH volume (van Straaten et al., 2006). Therefore, computational and visual metrics of WMH severity are complementary metrics which can provide additional insight.

### 4.3 Associations between tracer kinetic parameters and age

We found *PS* and *v_P_* declined weakly with age in GM, WM and WMH, Gd concentration in the vascular input functions and tracer concentration curves for GM, WM and WMH also tended to be lower across time. Peak concentration reflects intravascular contrast (Wardlaw et al., 2017) and is highly dependent on acquisition time. However, consistently higher Gd concentrations at later timepoints may be due to higher vascular surface area in younger patients, potentially contributing to the observed decline in *PS* with age in this population. Specifically, younger patients with a stroke and established WMH, required for inclusion, may have inherently worse vascular state than an older person with a stroke and similar WMH severity. Age can therefore be a major confounder when a disease must be present in a study. Several previous studies have found older age to be associated with markers of increased permeability (Farrall and Wardlaw, 2009, Wardlaw et al., 2003), including CSF:plasma albumin ratio (Castellazzi et al., 2020) and DCE-MRI semi-quantitative (Wardlaw et al., 2017) approaches. Taheri et al did not find a correlation between age and *K_i_* or CSF:plasma albumin ratio in patients with suspected vascular cognitive impairment (Taheri et al., 2011). Montagne et al found *K^Trans^* in the hippocampus increased with age but no associations between age and permeability in a restricted sample of white matter in the same subjects (Montagne et al., 2015). Increased *K^Trans^* in the hippocampus and parahippocampal gyrus has also been reported in patients with early cognitive dysfunction relative to controls and adjusting for age, but found no association with age (Nation et al., 2019). Several papers compare to age-matched controls or include age as a covariate in their statistical analysis but do not report regression coefficients for age (Li et al., 2017, van de Haar et al., 2016a, van de Haar et al., 2016b, Zhang et al., 2019, Zhang et al., 2017). As such, the associations between tracer kinetic parameters and age in SVD populations have been under explored. In older patients, perforating arterioles and capillaries become sparse with fewer branches resulting in a reduced vascular surface area (Bailey et al., 2004), which would be consistent with reduced *v_P_*. As *PS* is the product of vascular surface area and permeability, reductions in *PS* with age may be explained by vessel density and size changes; indeed this has been proposed as an explanation for the apparent higher values of *PS* in GM vs. WM (Østergaard et al., 2016, Varatharaj et al., 2019). Such considerations may therefore affect the interpretation of *PS* values, particularly in WMH, where they may be less indicative of permeability than decreasing vascular surface area. Current techniques do not allow reliable measurement of vessel size and density *in vivo*, indicating an urgent need for further preclinical, post-mortem or retinal studies to better account for vessel sparsity and surface area. Existing comorbidities also complicate interpretation of age-related effects in SVD patients (Wardlaw et al., 2019).

### 4.4 Associations between tracer kinetic parameters and hypertension

Patients with hypertension had decreased *v_P_* in WM, GM and WMH, and increased *PS* in recent stroke lesions. Hypertension is a SVD risk factor (Yakushiji et al., 2014), linked to WMH burden and, via endothelial impairment, BBB dysfunction, particularly when uncontrolled (Meissner, 2016). Over time, high blood pressure is thought to lead to stiffening of the arteries, reduced vasoreactivity and impaired autoregulation of cerebral blood flow (Meissner, 2016). Regional decreases in cerebral blood flow are reported in older patients with hypertension (Beason-Held et al., 2007) and WMH (Shi et al., 2016). Hypertension related vascular changes may therefore contribute to lower *v_P_* via reductions in blood flow or potentially underlying vascular changes.

### 4.5 Strengths/limitations

The strengths of this study include the large, well-characterised cohort of patients scanned on a single scanner, the largest such sample to date, and quantitative measurement of physiological parameters (*PS* and *v_P_*) closely following consensus recommendations (Thrippleton et al., 2019), adjusted for key covariates. Limitations include the methodological shortcomings of existing methods for acquiring and measuring BBB permeability in patients *in vivo*, slightly lower temporal resolution and longer acquisition duration than recommended (Thrippleton et al., 2019). Use of DCE-MRI to measure subtle BBB leakage, as thought to occur in SVD, remains challenging because of the low-level BBB leakage and comparatively large molecular size of Gadolinium based contrast agents (Nitta et al., 2003, Shao et al., 2020). Gadolinium use also limits inclusion of patients with impaired kidney function. While other approaches exist, including multiple flip angle multi-echo and diffusion-weighted arterial spin labelling quantification of transendothelial water exchange (Dickie et al., 2020), [68Gd]EDTA positron emission tomography (PET) (Schlageter et al., 1987), CSF albumin measurement (Janelidze et al., 2017) etc, these also have inherent limitations, specifically: the BBB is permeable to water via various mechanisms hence water exchange may not reflect pathological leakage, and is also very difficult to measure (Dickie et al., 2020); CSF markers do not provide regional specificity to BBB permeability and are influenced by CSF flow (Janelidze et al., 2017); PET and CT involve exposure to ionising radiation. Careful application of tissue-based regions of interest helps reduce noise and partial volume effects, however in patients with higher WMH burden, the residual volumes of WM and GM may be small and subject to greater contamination potentially skewing associations. Lastly, while Patlak is currently considered the best of the available models for tracer kinetic analysis of low level BBB leakage (Heye et al., 2016), all such models make assumptions regarding the physiological state and real deviations from these may confound the measurements and cannot currently be quantified.

### 4.6 Future directions

Further validation and development of existing and novel techniques to quantify BBB integrity in-vivo are required to broaden usage and provide a basis for further developments (Thrippleton et al., 2019). In particular, methodological development is required to account for differences in vascular surface area which may effect estimates of *PS*. A surrogate metric relating to surface area, such as *v_B_* (Varatharaj et al., 2019), could alternatively be included as a predictor variable. However, validation would be required to determine how representative such metrics are of the vascular surface area. In-silico methods are also a promising approach to evaluate the influence of different noise sources, acquisition, and analysis strategies at minimal cost (Bernal et al., 2021, Manning et al., 2021). While promising non-contrast based imaging MRI-based techniques, including diffusion-weighted arterial spin labelling, require further development, particularly to improve the limited SNR in white matter. Lastly, regional analyses may mask local differences, WMH may vary in their constituent tissue characteristics and underlying vascular (dys)function depending on their age and stage of evolution. Longitudinal studies at higher magnetic field strengths may allow systematic comparisons with structural neuroimaging data to investigate how local BBB permeability relates to visible pathologies (Clancy et al., 2021).

### 4.7 Conclusion

In conclusion, we have shown quantitative measurement of BBB integrity using tracer kinetic modelling reveals associations between microvascular integrity and SVD. To our knowledge our study is the largest in which such whole brain measures have been obtained in a population with a high prevalence of SVD. The relationship between clinically relevant markers of SVD and BBB permeability is complex due to the heterogeneity of the condition and methodological challenges. Despite this, the emerging associations between BBB impairment and clinically relevant parameters indicate that it is an important mechanism for further study to better understand the pathophysiology of SVD and as a potential target for development of novel treatments.

## Declaration of Competing Interest

The authors declare that they have no known competing financial interests or personal relationships that could have appeared to influence the work reported in this paper.
