# Supplementary material for: Tracer kinetic assessment of blood–brain barrier leakage and blood volume in cerebral small vessel disease: Associations with disease burden and vascular risk factors
Source: Neuroimage Clin. 2021 Nov 17;32:102883. doi: 10.1016/j.nicl.2021.102883 (PMC8607271; doi:10.1016/j.nicl.2021.102883)
Supplement: Supplementary data 1 [file mmc1.docx]

**Supplementary Table 1:** Permeability surface area (*PS*, 10^-4^ min^-1^) against age (years), percentage white matter hyperintensity in intracranial volume (% WMH in ICV), stroke subtype, mean arterial pressure (mmHg), hypertension status, pulse pressure (mmHg) and smoking status in each tissue type of interest. (WM=white matter, CI=confidence interval, MAP=mean arterial pressure).

|  | **Tissue *PS* associations using %WMH in ICV** | | | |
| --- | --- | --- | --- | --- |
| **Tissue** | **Variable** | **B coefficient** | **95% CI** | **p-value** |
| Normal appearing WM | Age | -0.037 | -0.062 to -0.011 | *0.004* |
|  | % WMH in ICV | 0.069 | -0.092 to 0.229 | 0.401 |
|  | Stroke subtype | -0.346 | -0.815 to 0.124 | 0.148 |
|  | MAP | -0.007 | -0.024 to 0.011 | 0.450 |
|  | Hypertension | 0.437 | -0.120 to 0.994 | 0.123 |
|  | Pulse pressure | 0.004 | -0.010 to 0.017 | 0.578 |
|  | Smoking status | -0.389 | -0.910 to 0.133 | 0.143 |
| WM Hyperintensities | Age | -0.029 | -0.056 to -0.002 | *0.037* |
|  | % WMH in ICV | 0.091 | -0.083 to 0.264 | 0.303 |
|  | Stroke subtype | -0.018 | -0.525 to 0.489 | 0.945 |
|  | MAP | 0.002 | -0.017 to 0.021 | 0.865 |
|  | Hypertension | 0.304 | -0.300 to 0.908 | 0.322 |
|  | Pulse pressure | 0.001 | -0.013 to 0.016 | 0.859 |
|  | Smoking status | -0.027 | -0.592 to 0.539 | 0.926 |
| Grey matter | Age | -0.039 | -0.063 to -0.016 | *0.001* |
|  | % WMH in ICV | 0.114 | -0.037 to 0.266 | 0.138 |
|  | Stroke subtype | -0.243 | -0.685 to 0.200 | 0.280 |
|  | MAP | -0.004 | -0.021 to 0.012 | 0.628 |
|  | Hypertension | 0.217 | -0.308 to 0.741 | 0.417 |
|  | Pulse pressure | 0.010 | -0.003 to 0.023 | 0.123 |
|  | Smoking status | 0.166 | -0.326 to 0.658 | 0.507 |
| Recent stroke lesion | Age | 0.024 | -0.062 to 0.109 | 0.587 |
|  | % WMH in ICV | -0.146 | -0.677 to 0.385 | 0.588 |
|  | Stroke subtype | -1.992 | -3.584 to -0.400 | *0.015* |
|  | MAP | 0.026 | -0.034 to 0.085 | 0.397 |
|  | Hypertension | 2.203 | 0.206 to 4.199 | *0.031* |
|  | Pulse pressure | -0.018 | -0.063 to 0.026 | 0.421 |
|  | Smoking status | 0.776 | -0.958 to 2.510 | 0.378 |
|  |  |  |  |  |

**Supplementary Table 2**: Plasma volume fraction (*v_P_*, 10^-2^) against age (years), percentage white matter hyperintensity in intracranial volume (% WMH in ICV), stroke subtype, mean arterial pressure (mmHg), hypertension status, pulse pressure (mmHg) and smoking status in each tissue type of interest. (WM=white matter, CI=confidence interval, MAP=mean arterial pressure).

|  | **Tissue v_P_ associations using %WMH in ICV** | | | |
| --- | --- | --- | --- | --- |
| **Tissue** | **Variable** | **B coefficient** | **95% CI** | **p-value** |
| Normal appearing WM | Age | -0.008 | -0.013 to -0.002 | *0.005* |
|  | % WMH in ICV | 0.041 | 0.006 to 0.076 | *0.021* |
|  | Stroke subtype | 0.065 | -0.037 to 0.166 | 0.211 |
|  | MAP | 0.001 | -0.003 to 0.005 | 0.597 |
|  | Hypertension | -0.166 | -0.287 to -0.046 | *0.007* |
|  | Pulse pressure | -0.000 | -0.003 to 0.003 | 0.840 |
|  | Smoking status | -0.104 | -0.217 to 0.009 | *0.072* |
| WM Hyperintensities | Age | -0.010 | -0.018 to -0.002 | *0.011* |
|  | % WMH in ICV | -0.066 | -0.116 to -0.015 | *0.011* |
|  | Stroke subtype | 0.054 | -0.094 to 0.202 | 0.472 |
|  | MAP | 0.001 | -0.004 to 0.007 | 0.649 |
|  | Hypertension | -0.176 | -0.352 to 0.000 | *0.050* |
|  | Pulse pressure | -0.001 | -0.005 to 0.004 | 0.757 |
|  | Smoking status | -0.011 | -0.176 to 0.154 | 0.899 |
| Grey matter | Age | -0.009 | -0.014 to -0.003 | *0.005* |
|  | % WMH in ICV | 0.029 | -0.009 to 0.067 | 0.137 |
|  | Stroke subtype | 0.071 | -0.041 to 0.183 | 0.212 |
|  | MAP | -0.000 | -0.004 to 0.004 | 0.998 |
|  | Hypertension | -0.153 | -0.285 to -0.020 | *0.024* |
|  | Pulse pressure | -0.001 | -0.004 to 0.002 | 0.616 |
|  | Smoking status | -0.137 | -0.262 to -0.013 | *0.031* |
| Recent stroke lesion | Age | -0.001 | -0.012 to 0.009 | 0.795 |
|  | % WMH in ICV | -0.017 | -0.085 to 0.050 | 0.609 |
|  | Stroke subtype | -0.143 | -0.345 to 0.060 | 0.165 |
|  | MAP | 0.001 | -0.007 to 0.009 | 0.780 |
|  | Hypertension | -0.163 | -0.417 to 0.091 | 0.207 |
|  | Pulse pressure | 0.001 | -0.004 to 0.007 | 0.608 |
|  | Smoking status | 0.086 | -0.134 to 0.306 | 0.441 |

**Supplementary Figure 1**: Scatter plots of permeability surface area (*PS*, 10^-4^ min^-1^) against slope of the enhancement curve (% min^-1^). (WM: normal appearing white matter, WMH: white matter hyperintensities, GM: grey matter and RSL: recent stroke lesion).


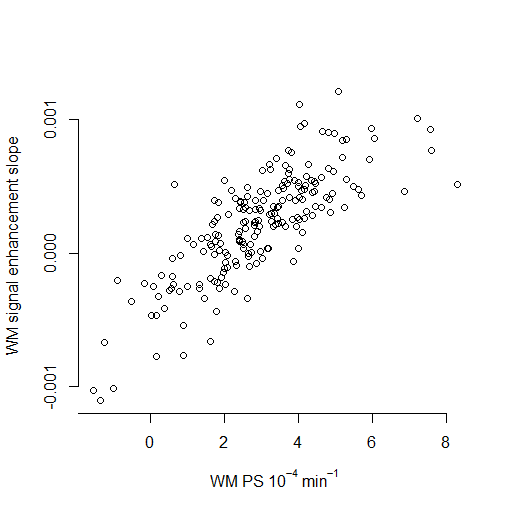

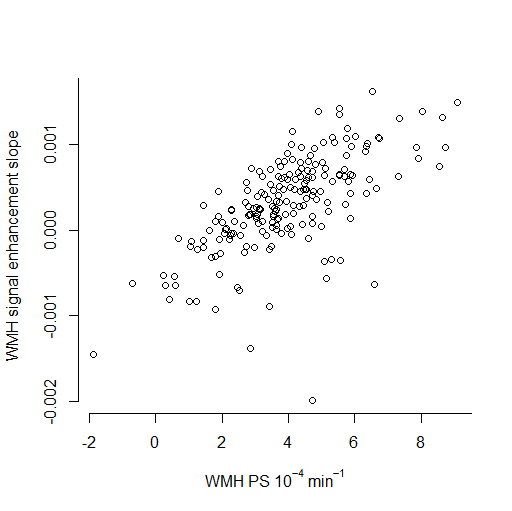

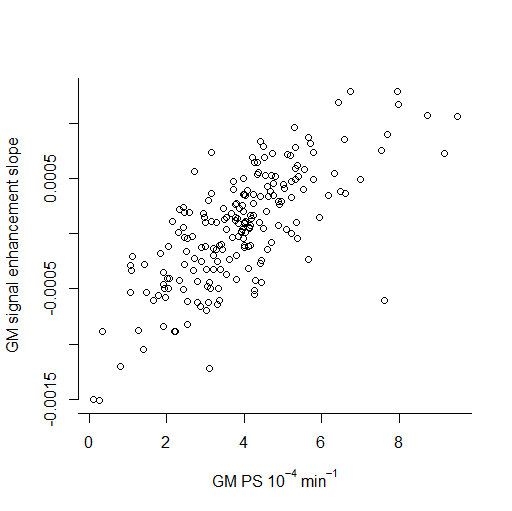

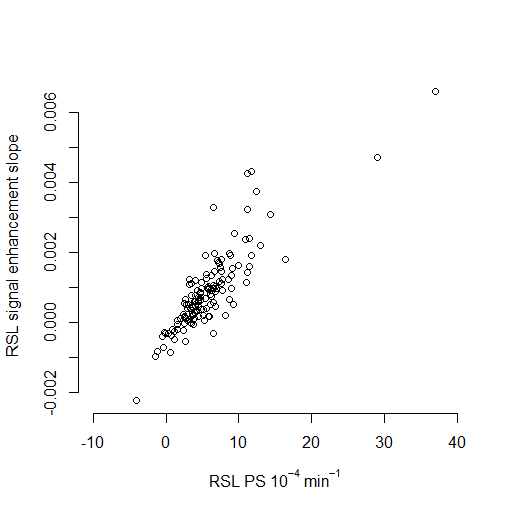


Grey matter

Recent stroke lesion

White matter

White matter hyperintensities

*PS against enhancement slope by tissue type*

WM signal enhancement (% min^-1^)

WMH signal enhancement (% min^-1^)

GM signal enhancement (% min^-1^)

RSL signal enhancement (% min^-1^)

WM PS (10^-4^min^-1^)

RSL PS (10^-4^min^-1^)

GM PS (10^-4^min^-1^)

WMH PS (10^-4^min^-1^)

-0.10

0.00

0.10

-0.10

0.00

0.10

-0.20

-0.15

-0.05

0.05

0.20

0.40

0.60

0.00

-0.20
